# Bioactive Compounds and Quality Evaluation of Red-Pigmented Rice Processed by Germination and Roasting

**DOI:** 10.3390/foods11182735

**Published:** 2022-09-06

**Authors:** Soo-Ji Kang, Su-Yeon Jeong, Mohammad Zahirul Islam, Bo-Kyung Shin, Young Jin Park, Jae Kwang Kim, Young-Tack Lee, Jong-Hun Lee

**Affiliations:** 1 Department of Food Science and Biotechnology, Gachon University, Seongnam 13120, Korea; 2 Division of Life Sciences, College of Life Sciences and Bioengineering, Incheon National University, Incheon 22012, Korea

**Keywords:** red-pigmented rice, germination, roasting, bioactive compounds, quality

## Abstract

Red-pigmented rice was germinated and processed to develop germinated red rice tea, and the changes in physicochemical, bioactive, and microbial properties due to germination and roasting were investigated. The moisture and crude ash contents of red rice decreased after germination and roasting. Crude protein and crude fat contents increased after germination but slightly decreased after roasting. Total phenolics, flavonoids, and antioxidant activities (DPPH and ABTS radical scavenging activities) increased following germination and heat treatment. However, the increased levels of γ-amino butyric acid after germination significantly decreased during the subsequent roasting step. In addition, total bacteria, yeast, and mold counts increased during the germination process but decreased after heat treatment as compared to those in the original grain; *Escherichia coli* was not detected. Therefore, germination and subsequent roasting could effectively enhance the contents of the most bioactive compounds and maintain microbial stability in red-pigmented rice.

## 1. Introduction

Rice (*Oryza sativa* L.) is one of the most important cereal crops worldwide and a popular staple food, especially in Asia. Itis predominantly consumed in milled form as white rice, followed by brown rice. There are also colored rice genotypes that exhibit black, purple, or red pigmentation. Pigments, expressed in the bran layers of rice kernels, are a mixture of anthocyanin [1,2,3]. Red rice is reported to be rich in nutritional and biofunctional components such as proanthocyanidins, γ-oryzanol, γ-aminobutyric acid (GABA), dietary fibers, vitamins, and minerals, as compared with common brown rice [4,5]. Higher flavonoid and polyphenol contents are often linked to higher antioxidant activity [6]. Pigmented rice cultivars have higher anthocyanin, phenolic compounds, and bioactive compounds than non-pigmented rice cultivars, and their consumption has been associated with decreased risk of diabetes, cardiovascular disease, and cancer [1,7,8].

Germination is known to be an effective way to improve grain quality, as it softens the kernel structure and improves nutritional value [9]. Germinated brown rice (GBR) has recently gained popularity in Asia. Germinated rice contains higher quantities of phytochemicals, including GABA, tocopherol, 𝛾-oryzanol, and phenolic compounds than non-germinated rice [10,11]. Germinated grain products have been used as new ingredients in the food industry for improving flavor, taste, mineral absorption, and nutritional value. Roasting is a common processing technology that enhances several properties such as color, aroma, flavor, and textural property, and has been widely used in whole grain rice products [12]. Moreover, it can remove some undesirable volatile organic compounds that might be formed during germination process [13]. However, thus far, there is very limited research available on the quality characteristics of red-pigmented rice processed by germination and roasting.

Therefore, this study was conducted to examine the effects of germination and roasting on the physicochemical, bioactive, and microbiological properties of red-pigmented rice and to determine the possibility of using the product as germinated red rice tea.

## 2. Materials and Methods

### 2.1. Red-Pigmented Rice and Germination Process

The red-pigmented rice used in this experiment was harvested and collected from Cheongwon, Korea, in 2019. Dehulled red rice was cleaned manually, and defective grains were removed. Red rice was soaked in distilled water at 20 °C for 24 h, and the soaked grain were transferred to a seed germinator (Dasol Scientific Co., Ltd., Hwaseong, Korea) where they were allowed to germinate at 25 °C and 90% humidity for 30 h. Germinated, red-pigmented rice grains were dried at 55 °C for 18 h in a dryer.

### 2.2. Processing of Germinated Rice and Red- Pigmented Rice Tea Production

**Figure 1:**
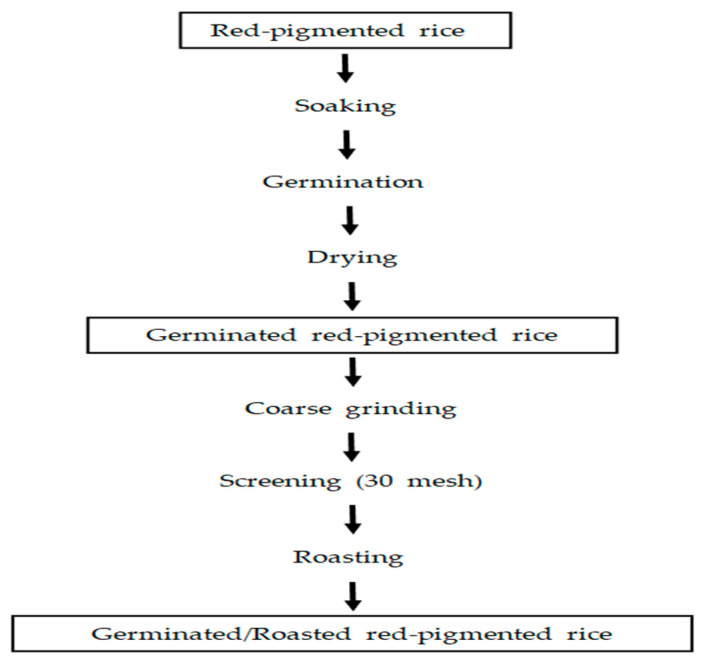
Procedure for producing germinated red-pigmented rice tea.

Dry, germinated, red-pigmented rice was crushed using a roll crusher (Poongin Co., Pyeongtaek, Korea) and roasted at 180 °C for 6 min using a grain roaster. The processing procedure for the preparation of germinated red rice tea is shown in Figure 1. Red-pigmented rice, germinated red-pigmented rice after drying, and roasted red-pigmented rice were used as samples after grinding using a grinder (Nutri Mill, UT, USA).

### 2.3. Proximate Analysis

Moisture, crude protein, crude fat, and crude ash contents were measured according to AACC approved methods 44-16, 46-13, 30-10, and 08-01, respectively [14]. Total carbohydrate was calculated as: % Carbohydrate = 100% − (% crude protein + % crude fat + % crude ash).

### 2.4. Color Measurement

A color difference meter (CR-400, Konica Minolta Sensing, Inc., Japan) was used to measure L*, a*, and b*. The parameters L*, (+)a*, and (+)b* indicate brightness or lightness, redness, and yellowness, respectively.

### 2.5. GABA Content

GABA was extracted according to the method described by Park et al. [15]. GABA content was determined on a GCMS-QP2010 Ultra System (Shimadzu, Japan) equipped with an auto sampler (AOC-20i, Shimadzu, Kyoto, Japan) and a DB-5 column (30 m length, 0.25 mm diameter, 1 μm thickness, Agilent Technologies, USA). Each sample was injected at 1 μL volume and a split ratio of 10:1. The injection, ion source, and interface temperatures were 280 °C, 200 °C, and 280 °C, respectively. Helium was used as the carrier gas (99.99%), and the column flow rate was 1.10 mL/min. Retention times (RT) and mass spectra of all samples were analyzed following comparison with the corresponding values for the standard sample.

### 2.6. Total Phenolic Content

The total phenolic content was determined according to the method described by Lamuela-Raventós [16], with slight modifications. Two grams of samples and distilled water (10 times) were mixed and extracted by stirring at 20 °C for 4 h. The samples were centrifuged for 30 min at 3500× *g*, and the supernatant was separated for analysis. Later, 0.8 mL of distilled water was added to 0.2 mL of extract sample along with 0.2 mL of Folin-Ciocalteu phenol reagent for 5 min at 20 °C. At the end of the reaction, the mixture was vortexed with 2 mL of 7% sodium carbonate (Na_2_CO_3_) solution for 1 h, and the absorbance was measured at 734 nm using a spectrophotometer (Karaltay Scientific Instruments, Beijing, China). A gallic acid standard curve prepared using 0–200 ppm concentrations was employed to calculate the total phenolic content.

### 2.7. Total Flavonoid Content

The total flavonoid content was measured with some modifications according to the method described by Pękal and Pyrzynska [17]. In brief, 0.5 mL of extract sample was mixed with 1.5 mL of ethanol, 0.1 mL of 10% (*w*/*v*) aluminum nitrate, 0.1 mL of potassium acetate (1 M), and 2.8 mL of distilled water in a test tube. The mixture was reacted for 40 min in the dark at 20 °C, before measuring the absorbance at 415 nm using a spectrophotometer. A quercetin standard curve in the concentration of 0–200 ppm was prepared and used to calculate the total flavonoid content.

### 2.8. In Vitro Antioxidant Activity

The radical scavenging activity of 2,2-diphenyl-1-picrylhydrazyl (DPPH) was measured using the method described by Islam et al. [18], with slight modifications. In brief, 0.2 mL of the sample extract was mixed with 0.8 mL of methanol solution containing DPPH (0.4 mM). The mixture was vigorously shaken for 10 s and left to stand for 10 min in the dark. Absorbance was measured at 525 nm wavelength using a spectrophotometer. The scavenging activity was determined according to the formula:

DPPH radical-scavenging activity (%) = (A − B)/A × 100

where A is the absorbance of the control and B is the absorbance of the sample extract.

The radical-scavenging activity of 2,2′-azinobis-(3-ethylbenzothiazoline-6-sulfonic acid) (ABTS) was measured using the method described by Re et al. [19] with slight modifications. The ABTS solution (1.9 mL) was mixed with 50 µL of standard or test extract; the sample was stored for 10 min in the dark at 20 °C before measuring its absorbance at 734 nm with a spectrophotometer. The absorbance of the ABTS^+•^ blank solution was also measured. The percentage of ABTS inhibition was calculated using the formula:

Inhibition of A_734_ (%) = (*1* − *A_S_/A_b_*) × 100, where A_S_ is the absorbance of the sample extract and A_b_ is the absorbance of the blank.

### 2.9. Microbial Analyses

Microbial counts for red-pigmented rice samples were enumerated using the standard plate count method [20]. Red-pigmented rice samples (25 g) were weighed into a stomacher bag (3M, St. Paul, MN, USA) containing 225 mL of sterile physiological saline solution and homogenized for 2 min using a paddle blender (IUL Instruments, Barcelona, Spain). Under aseptic conditions, 1 mL aliquots from the stomacher bag filtrate were serially diluted in sterile saline solution, and appropriately diluted solutions (0.1 mL) were plated in Petri dishes containing melted agar culture media and incubated at 37 °C for 24–48 h. Total aerobic bacteria, yeast, and mold were counted using plate count agar (PCA) and potato dextrose agar (PDA), respectively. For *Escherichia coli* detection, eosin-methylene blue agar was used as a selective enrichment medium (Difco, Becton Dickinson and Co., Sparks, NV, USA).

### 2.10. Statistical Analysis

Duncan’s multiple range test was conducted to analyze statistical significance between samples (*p* ≤ 0.05) using SPSS V.25 (SPSS Inc., Chicago, IL, USA).

## 3. Results and Discussion

### 3.1. Proximate Composition of Red-Pigmented Rice by Germination and Roasting

The proximate composition of the red-pigmented rice samples processed by germination and roasting is presented in Table 1. Raw red-pigmented rice had higher moisture content (12.67%) than the rice processed by germination (7.20%) and roasting (3.41%). Before processing, raw red-pigmented rice contained 8.69% crude protein, 2.36% fat, and 1.52% ash. After 30 h of germination, the crude protein of red rice increased to 9.51% and its crude fat content gradually increased to 3%. The increase in the crude protein and fat contents may be attributed to the decrease in carbohydrates used for respiration [21,22]. The crude ash content slightly reduced during germination and roasting, but there were no significant variations. Significantly higher total carbohydrate content was found in germinated and roasted red-pigmented rice.

### 3.2. Color of Red-Pigmented Rice Processed by Germination and Roasting

The color values of red-pigmented rice processed by germination and roasting are listed in Table 2. The L* value of raw red rice decreased after germination and then increased after roasting. The color of germinated red rice brightened again after roasting, owing to the exposure of the internal endosperm following breaking of kernels before roasting. The +a* value (8.75) of raw red rice was similar to that of germinated red rice but considerably decreased to 4.95 after roasting. The +b* value of raw red rice was 10.04 and changed to 9.07 following germination and 7.49 after roasting.

### 3.3. GABA Content of Red-Pigmented Rice after Germination and Roasting

**Figure 2:**
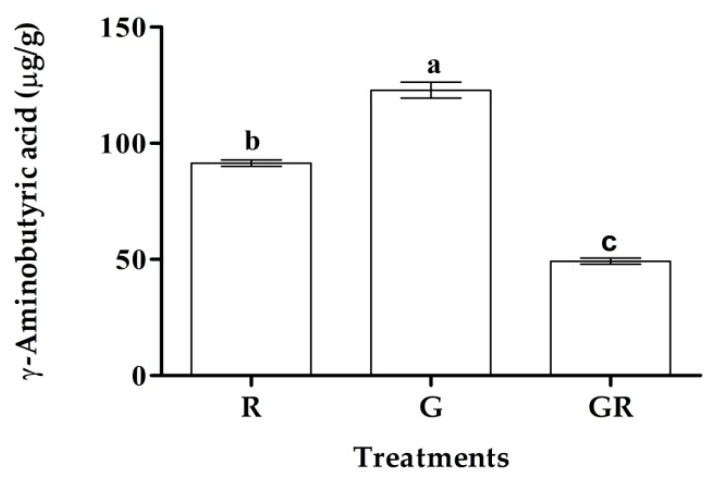
The GABA contents of red-pigmented rice in raw (R), germinated (G), and germinated and roasted (GR) conditions. Values expressed as means ± SD (*n* = 3).

The GABA contents of red-pigmented rice samples during processing are shown in Figure 2. The GABA content of raw red-pigmented rice was 91.44 μg/g; this value increased to 122.87 μg/g after 30 h germination. Changes in the GABA content of pigmented rice, as determined by high-performance liquid chromatography (HPLC), have been previously reported [23]. For example, the GABA content of red rice was shown to increase from 26.4 to 293.6 μg/g after germination, and that of black rice increased from 101.6 to 253 μg/g after germination. The increase in GABA content during germination is attributed to the rapid decarboxylation of glutamic acid, a GABA precursor, under environmental conditions during germination [24]. However, the GABA content significantly decreased during roasting. Previous reports have demonstrated the decrease in GABA content owing to roasting temperature and time [25,26], probably because GABA is lost via the action of the Maillard reaction following exposure to high temperatures.

### 3.4. Total Phenolic and Flavonoid Contents of Red-Pigmented Rice after Germination and Roasting

The total phenolic and flavonoid contents of red-pigmented rice processed by germination and roasting are shown in Table 3. The phenolic content of raw red-pigmented rice was 132.55 μg/g and increased to 156.35 μg/g after 30 h germination. The roasting process after germination further increased the phenolic content of rice. The high phenolic content of roasted, red-pigmented rice was probably attributed to the degradation of the interior tissue and rapid elution of phenolic chemicals during the roasting process. This result was similar to that previously reported where total phenolic content tended to increase with an increase in the roasting temperature and time of germinated rice tea [27]. Phenolics are representative antioxidants that eliminate radicals by donating hydrogen to alkyl radicals or alkyl peroxy radicals [28]. Phenolics have different health-promoting properties, including anti-oxidation of low-density-lipoprotein, cholesterol and liposomes [29], and prevention of colon cancer, and anti-inflammatory action [7].

The total flavonoid content increased from 22.89 μg/g for raw red-pigmented rice to 27.80 μg/g for germinated rice. With the support of acetyl coenzyme A ester, the phenylpropanoid metabolic pathway generated flavonoids in germinated rice [30]. Flavonoid levels increase owing to an up regulation in the expression of flavone synthesis genes before the completion of germination process [31]. The total flavonoid content of red-pigmented rice was the highest after roasting and reached 33.68 μg/g. Flavonoids are one of the most powerful antioxidant phenolic compounds in terms of elimination of oxygen from the body and exert anticancer, antitumor, anti-atherosclerotic, anti-thrombogenic, anti-osteoporotic, and antiviral effects. Moreover, these compounds alleviate inflammation, allergies, cardiovascular disease, and ischemia-reperfusion injury [7,8,32].

### 3.5. Antioxidant Activity of Red-Pigmented Rice after Germination and Roasting

The antioxidant activities of red-pigmented rice during germination and roasting are presented in Table 4. DPPH and ABTS assays are the most commonly used methods for measuring antioxidant activity [33,34]. Prior to germination, red-pigmented rice had DPPH and ABTS radical-scavenging activities of 50.48% and 71.01%, respectively. The DPPH and ABTS radical-scavenging activity of germinated, red-pigmented rice was increased by 1.41 and 1.03 times, respectively. Germination increases the free radical scavenging activity of brown rice [35,36] and pigmented rice [37]. The DPPH and ABTS radical-scavenging abilities were found to be the highest for red-pigmented rice after germination and the subsequent roasting step. In comparison to raw red-pigmented rice, the elevated value for DPPH was 1.54 times higher and 1.05 times higher for ABTS. This result is in line with that reported by Lee et al. [27] who showed that germinated rice tea exhibits a better antioxidant performance with an increase in the temperature and time of roasting. Studies have indicated that increasing polyphenol content boosts the DPPH and ABTS radical scavenging capabilities [38].

### 3.6. Microbial Growth in Red-Pigmented Rice after Germination and Roasting

The changes in the microbial counts of red-pigmented rice processed by germination and roasting are shown in Table 5. Prior to germination, raw red-pigmented rice had the lowest counts of total aerobic bacteria, yeast, and mold. The total aerobic plate counts in raw red-pigmented rice increased from 1.3 × 10^4^ to 2.5 × 10^7^ CFU/g after 30 h of germination, but significantly decreased to 1.7 × 10^4^ CFU/g after roasting. The increase in the total aerobic count of red-pigmented rice after germination was similar to that previously reported [39] in germinated brown rice, where the aerobic bacterial count was 3.9 log CFU/g at the beginning of germination and increased to 6.5 log CFU/g after 36 h of germination. The yeast and mold counts in red-pigmented rice also increased to 8.4 × 10^6^ CFU/g after germination and decreased to 7.0 × 10^3^ CFU/g through the roasting process, as did the total number of bacteria. No *E. coli* was detected in red-pigmented rice during processing. As microbial growth and off-flavor generation increase during germination [39,40], pre- or post-treatment may be necessary to control these factors.

## 4. Conclusions

This study examined the impact of germination and roasting on the bioactive components and grain qualities of red-pigmented rice. Germination and roasting significantly increased the abundance of bioactive compounds such as phenolics and flavonoids, subsequently improving the antioxidant activities of the rice. The roasting process after germination also played an important role in maintaining microbial stability. Therefore, this research could contribute to the development of a unique red-pigmented rice tea by adopting an appropriate germination and roasting procedure.

## Figures and Tables

**Table 1:** Effects of germination and roasting on proximate composition (%) of red-pigmented rice.

	Moisture	Crude Protein	Crude Fat	Crude Ash	Total Carbohydrate
Red-pigmented rice (raw)	12.67 ± 0.19a ^z^	8.69 ± 0.18b	2.36 ± 0.11b	1.52 ± 0.19a	87.43 ± 0.52a
Germinated	7.20 ± 0.06b	9.51 ± 0.41a	3.00 ± 0.13a	1.45 ± 0.04a	86.04 ± 0.42b
Germinated & roasted	3.41 ± 0.13c	9.36 ± 0.31a	2.87 ± 0.05a	1.38 ± 0.05a	86.39 ± 0.59b

Values are on a dry basis, except for moisture. Values expressed as means± SD (*n* = 3). ^z^ Mean values within a column by the same letter are not significantly different (*p* ≤ 0.05).

**Table 2:** Effects of germination and roasting on color values of red-pigmented rice.

	Color Values		
	Lightness (L*)	Redness (+a*)	Yellowness (+b*)
Red-pigmented rice (raw)	44.05 ± 1.13b ^z^	8.75 ± 0.08a	10.04 ± 0.60a
Germinated	42.48 ± 0.64c	8.78 ± 0.78a	9.07 ± 0.51b
Germinated & roasted	50.02 ± 1.61a	4.95 ± 0.33b	7.49 ± 0.06c

Values expressed as means ± SD (*n* = 3). ^z^ Mean values within a column by the same letter are not significantly different (*p* ≤ 0.05).

**Table 3:** Effects of germination and roasting on total phenolic and flavonoid contents of red-pigmented rice.

	Total Phenolic Content (μg/g)	Total Flavonoid Content (μg/g)
Red-pigmented rice (raw)	132.55 ± 2.26b ^z^	22.89 ± 2.56c
Germinated	156.35 ± 2.55a	27.80 ± 1.19b
Germinated & roasted	161.00 ± 3.19a	33.68 ± 2.24a

Values expressed as means ± SD (*n* = 3). ^z^ Mean values within a column by the same letter are not significantly different (*p* ≤ 0.05).

**Table 4:** Effects of germination and roasting on *in vitro* antioxidant activity of red-pigmented rice.

	Antioxidant Activity	
	DPPH Radical- Scavenging Activity (%)	ABTS Radical- Scavenging Activity (%)
Red-pigmented rice (raw)	50.48 ± 2.71c ^z^	77.01 ± 0.85c
Germinated	71.09 ± 2.75b	79.00 ± 0.24b
Germinated & roasted	77.91 ± 1.16a	81.11 ± 1.14a

Values expressed as means ± SD (*n* = 3). ^z^ Mean values within a column by the same letter are not significantly different (*p* ≤ 0.05).

**Table 5:** Effects of germination and roasting on microbial counts of red-pigmented rice.

	Total Bacteria (CFU/g)	Yeast &Mold (CFU/g)	*E. coli* (CFU/g)
Red-pigmented rice (raw)	1.3 × 10^4^b ^z^	8.8 × 10^2^c	ND
Germinated	2.5 × 10^7^a	8.4 × 10^6^a	ND
Germinated & roasted	1.7 × 10^4^b	7.0 × 10^3^b	ND

Values expressed as means ± SD (*n* = 3). ^z^ Mean values within a column by the same letter are not significantly different (*p* ≤ 0.05). Note. ND, not detected; CFU, colony forming Unit.

## Data Availability

Not applicable.
